# Crystalline and magnetic properties of CoO nanoparticles locally investigated by using radioactive indium tracer

**DOI:** 10.1038/s41598-021-99810-y

**Published:** 2021-10-25

**Authors:** Renata V. Santos, Gabriel A. Cabrera-Pasca, Cleidilane S. Costa, Brianna Bosch-Santos, Larissa Otubo, Luciano F. D. Pereira, Bruno S. Correa, Fernando B. Effenberger, Anastasia Burimova, Rafael S. Freitas, Artur W. Carbonari

**Affiliations:** 1 Programa de Pós-Graduação em Ciência e Engenharia de Materiais – PPGCEM, Universidade Federal do Pará, Ananindeua, PA 67130-660 Brazil; 2 Faculdade de Ciências Exatas e Tecnologia, Universidade Federal do Pará, Abaetetuba, PA 68440-000 Brazil; 3 Instituto de Pesquisas Energéticas e Nucleares, IPEN-CNEN/SP, 05508-000 São Paulo, SP Brazil; 4 Instituto de Física, Universidade de São Paulo, São Paulo, SP 05508-090 Brazil

**Keywords:** Nanoparticles, Magnetic properties and materials

## Abstract

We herein report a comprehensive investigation on the magnetic, structural, and electric properties of CoO nanoparticles with different sizes by local inspection through hyperfine interactions measured in a wide range of temperatures (10–670 K) by using radioactive $^{111}$In($^{111}$Cd) tracers with the perturbed angular correlations technique. Small cobalt oxide nanoparticles with the characteristic size of 6.5 nm have been prepared by the wet chemical route that turned out to be essential to incorporate radioactivity tracers during nucleation and growth of the particles. Nanocrystalline samples with 22.1 nm size were obtained by thermal treatments under low pressure of helium at 670 K. The hyperfine data were correlated with X-ray diffraction, ZFC–FC magnetic measurements, and transmission electron microscopy to describe the structure, magnetic properties, size, and shape of samples. An analysis of the temperature evolution of hyperfine parameters revealed that the structural distortion and the magnetic disorder in the core and on the surface layer play an important role in the magnetic behavior of CoO nanoparticles.

## Introduction

Due to their great potential for application in new technological devices, magnetic nanoparticles (NPs) have captured the attention of researchers in several areas of scientific knowledge, and are being studied with increasing interest, promoting mainly the investigation of new synthesis routes and characterization methodology with the purpose to improve their performance^1–3^. For example, magnetic NPs can be applied in the production of ferrofluids, biosensors, battery materials, catalysts, drug delivery, magnetic resonance contrast agents, etc.^3–7^ All these applications are possible because NPs have shown a chemical stability and a change in the behavior of their magnetic properties. Particularly, NPs of magnetic transition metals are interesting because they present unique physical properties at the nanometric scales that differ drastically when compared to those of large particles^8–10^.

The area/volume ratio is large in NPs and increases when their size decreases enhancing the influence of surface layer on their physical properties. Therefore, local techniques sensitive to different regions within an atomic scale are necessary to perform a detailed investigation and characterization of NPs^11–14^. The better understanding of local correlations is expected to boost the development of new technological applications.

In order to have an adequate magnetic characterization of magnetic NPs, knowing their magnetic structure, transition temperature and the degree of crystallinity is necessary because these properties play an important role in the occurrence of magnetism. Regarding the magnetic phase transitions of nanoparticles, blocking temperature ($T_B$) often hinders the determination of the ordering temperature. $T_B$ is a signature of the particle size reduction, typically extracted from the zero field cooling and field cooling (ZFC–FC) curves of magnetic measurements, with timescale around milliseconds (ms).

There is a probability that the magnetization of small magnetic NPs flips to the opposite direction at a certain temperature. The mean time between two flips is called the Néel relaxation time ($\tau _N$). Considering that the magnetization in a magnetic nanoparticle is measured with a measurement time $\tau _m$, then if in one experiment $\tau _m \gg \tau _N,$ the nanoparticle magnetization will flip several times during the measurement and the measured magnetization will average to zero. On the other hand, if in another experiment $\tau _m \ll \tau _N$, the magnetization will not flip during the measurement, and the measured magnetization will be what the instantaneous magnetization was at the beginning of the measurement. The first experiment indicates that the nanoparticle would be in the superparamagnetic state, whereas in the second experiment it will be blocked in its initial state. Therefore, the state of the nanoparticle (superparamagnetic or blocked) depends on the measurement time that is characteristic of each experimental technique. A transition between superparamagnetic and blocked state occurs when $\tau _m = \tau _N$^15–17^.

Hence, to inspect the ordering temperature in magnetic nanoparticles, an experimental technique with a timescale around nanoseconds, which is much smaller than, for example, AC susceptibility measurements with a timescale around ms, can be useful. Hyperfine interaction techniques are suitable to accurately determine the transition temperatures and also allow characterizing short-range magnetic interactions^14,18^. In addition, the local techniques are very efficient, because they also allow to determine the crystallinity, the presence of spurious phases, and local crystal distortions which sometimes are imperceptible to X-ray diffraction since the particles are very small.

The perturbed angular correlation (PAC) spectroscopy is a non-resonance hyperfine interaction technique that uses radioisotopes as probes^19^. These probe nuclei are added to the nanoparticles during nucleation and growth syntheses in extremely low quantities (fractions of ppm) acting as radioactive tracers sensing different regions inside the nanoparticles^20,21^. Moreover, local techniques based on hyperfine interactions have been applied to numerous nanoparticles and corresponding results have been very important to understand the magnetic properties and local crystalline structure. Among them, we can mention the Mossbauer spectroscopy in $\hbox {Fe}_3 \hbox {O}_4$, $\alpha$-$\hbox {Fe}_2 \hbox {O}_3$, FePt nanoparticles among others^18,22,23^. In addition, Nuclear Magnetic Resonance (NMR) and muon spin rotation have been used to study the magnetic and structural properties in different types of nanoparticles^24–26^.

Cobalt monoxide (CoO) NPs have attracted much attention due to their numerous technological applications directly associated with chemical, structural and magnetic properties. In the nanometric scale, the CoO can be crystallized in the rock-salt cubic phase (space group $Fm{\bar{3}}m$) or the hexagonal wurtzite type (space group *P*63*mc*)^27–29^. Anomalous magnetic behavior has been observed in rock-salt type CoO NPs, *e.g.*, a ferromagnetic ordering in CoO nanocrystals with size smaller than 10-20 nm. At first, this ferromagnetic behavior was attributed to a decompensation of spins on the surface of very small CoO nanoparticles indicating that the surface spins play a significant role as suggested by Néel and discussed in works of NiO and MnO^30–33^. However, in the recent reports, the origin of ferromagnetic behavior at room temperature became a matter of debate^27,34^. In contrast, it is well known that the bulk fcc CoO has an antiferromagnetic behavior with a well-established Neél temperature around 292 K. On the other hand, the physical properties of the hcp-wurtzite phase have been the subject of recent studies, where the antiferromagnetic ordering in NPs with an average size of 45 nm was observed around $T_N$ $\sim$ 245 K^34^.

The main purpose of the work here reported is the investigation of the local electronic and magnetic properties, to determine the magnetic phase transition and the possible existence of local distortions in CoO nanoparticles by measuring hyperfine interactions by PAC spectroscopy. Samples of CoO NPs were synthesized by a wet chemical route, the thermal decomposition, which offers several advantages such as the control and low dispersion of particle size and allows the possibility of incorporating radioactive tracers—in the present work, the $^{111}$In nuclei—during the synthesis process. The hyperfine measurements were carried out in a wide range of temperatures in order to understand the structural and magnetic local properties. Additionally, the local properties were correlated with X-ray diffraction (XRD) and ZFC–FC magnetic measurements. The NP shape and size are described by high-resolution transmission electron microscopy (HRTEM).

## Results

A brief description of samples is necessary to follow the discussion of results. Two types of samples (hereafter named S1 and S2) are parts of the same synthesized material. S1 was characterized by XRD, TEM, and magnetizaton measurements just after synthesis, whereas S2 was characterized by the same techniques after annealing, which was carried out during PAC measurements at temperatures in the range from 295 K to 670 K. However, before this annealing, PAC measurements were performed in S2 (also just after synthesis) at temperatures below 295 K, whose results are then representative of S1 and could be correlated with their results of characterization.

**Figure 1:**
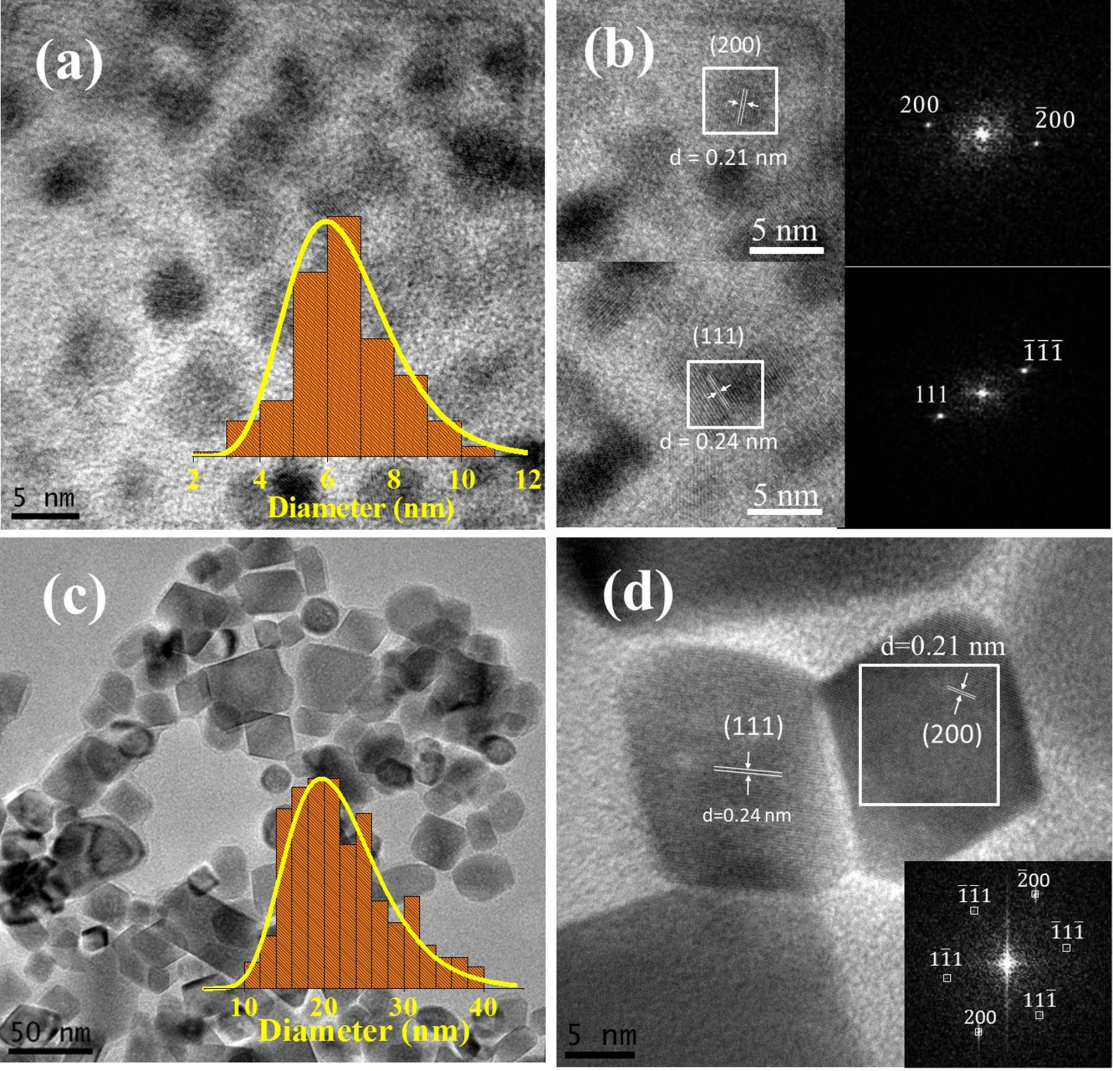
TEM images of CoO samples S1 (**a**) and S2 (**c**). The insets in (**a**) and (**c**) display the log-normal size distribution of particles. High-resolution (HRTEM) images of S1 (**b**) and S2 (**d**) revealing the regular arrangement of lattice fringes. The corresponding fast Fourier transform (FFT) of images are displayed in the insets in (**b**) and (**d**) and show the spacing of 0.24 nm and 0.21 nm at [111] and [200] orientations of CoO phase, respectively

TEM images of the samples S1 and S2 are displayed in Fig. 1 and show that S1 is composed by spherically-shaped particles with log-normal size distribution and mean diameter $< D_1 > = 6.5 \pm 1.6$ nm. The S2 particles was found to consist of non-uniformly shaped particles whose size also obeyed Galton’s distribution parameterized by $< D_2 > = 22.1 \pm 6.3$ nm.

High-resolution transmission electron microscopy (HRTEM) images displayed in Fig. 1b and d show the interplanar distance and reveal the [111] and [200] orientations of CoO phase in S1 and S2 nanocrystals. Distinctive sets of fringes can be unambiguously identified in some particles with (111) and others with (200) orientation planes. The clear and single type of lattice fringes free from dislocations and stacking faults indicate a single-crystalline nature.

XRD patterns of S1 and S2 samples are displayed in Fig. S1 in the Electronic Supplementary Information (ESI) and for both samples the main peaks (111), (200), (220), (311), (222) and (400) of Bragg planes are consistent with fcc-CoO structure phase. A single phase for the as-prepared S1 sample is observed, which corresponds to the fcc structure of CoO with the lattice parameter of 4.2665 Å. S2 sample presents two phases. The main phase (around 88.7 %) corresponds to the fcc structure with the lattice parameter of 4.2676 Å. The minority phase (around 11.3 %) corresponds to the hcp structure of Co with lattice parameters *a* = 2.5054 Åand *c* = 4.0893 Å. This metallic phase is assumed to be formed during the thermal treatment, which induces the oxide reduction^35,36^. The Scherrer equation was used in order to obtain the crystallite average size, which are found to be $\sim$ 7 nm for S1 and $\sim$ 20 nm for S2, both values are in total agreement with the TEM results shown above.

**Figure 2:**
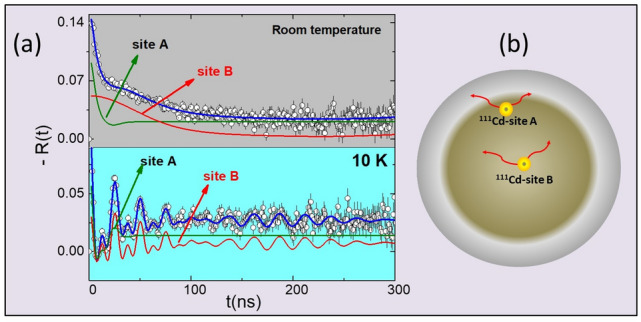
(**a**) Spin rotation spectra measured with $^{111}$Cd in S2 sample at room temperature (top) and 10 K (bottom). (**b**) Schematic drawing of the spherical particle displaying the positions of $^{111}$Cd probe nuclei (represented by small yellow spheres)

Spin rotation spectra *R*(*t*) of sample S2 measured with PAC spectroscopy at 10 K and at room temperature are displayed in Fig. 2. Spectrum taken at room temperature was fitted with a model considering that probe nuclei occupy two sites with different environments characterized by pure electric quadrupole interactions. The major fraction (henceforth referred as site A) with population $f_A = 65\%$, is characterized by a broadly distributed ($\delta _A = 80\%$) quadrupole frequency $\nu _{QA} = 62(4)$ MHz and asymmetry parameter $\eta$ = 0.35(2). Site A was ascribed to probe nuclei occupying the external layer of the nanoparticles where the reminiscent organic material covering the nanoparticles is present. A schematic drawing representing the layers of the particle is displayed in Fig. 2b. The minor fraction (henceforth referred as site B) with population $f_B = 35\%$ presents a less distributed ($\delta _B = 30\%$) and small quadrupole frequency $\nu _{QB} = 11(2)$ MHz with vanishing asymmetry parameter ($\eta _B = 0$). The small value of $\nu _Q$ and the fully symmetric site environment lead to the assignment of probe nuclei to substitutional cationic sites in a cubic structure, as expected for fcc-CoO. At room temperature, CoO is paramagnetic and, therefore, no dipole magnetic interactions are expected.

However, at 10 K, the magnetic interaction is present, and we have achieved an excellent fit of these spectra considering a model that employs two site fractions with combined interactions. The major fraction ($f_A = 65\%$), corresponding to probe nuclei at site A in the external layer of the particles, as described above, now is characterized by a quadrupole frequency $\nu _{QA} = 17(2)$ MHz with that employs two site fractions parameter ($\eta _A = 0)$ plus a magnetic frequency $\nu _{MA} = 37.1(2)$ MHz which corresponds to a magnetic hyperfine field $B_{hf} = 16.1(2)$ T . This site fraction presents a broad frequency distribution with $\delta _A = 40\%$ that was attributed to point defects near the surface, such as oxygen vacancies. The contribution of organic coating to the distortions at NP surface layer can not be discarded either.

On the other hand, at low temperatures, the site fraction B, with population $f_B = 35\%$, shows narrow frequency distribution attributed to cation substitution of probe nuclei at a regular site position. In addition, this site is characterized by a combined interaction of electric quadrupole with $\nu _QB = 12(2)$ MHz, $\eta _B$ = 0 and magnetic frequency $\nu _{MB} = 40.2(2)$ MHz with the angle between the directions of $V_{zz}$ and $B_{hf}$, $\beta = 27^{\circ }$. As mentioned before, because the quadrupole frequency is very small, site B was assigned to probe nuclei at substitutional Co sites in the cubic structure of CoO. This assignment is reinforced by the observed magnetic frequency for site B, corresponding to a magnetic hyperfine field $B_{hf} = 17.1(2)$ T, which totally agrees with 17.1(3) T reported by Rinneberg and Shirley^37^ for the magnetic hyperfine field at $^{111}$Cd nuclei in CoO. The mechanism of spin density transfer from Co ions to the s electrons of Cd occurs through the six oxygen nearest neighbors that octahedrally surround the Cd ion. This spin polarization from the nearest Co neighbors is transferred by orbital overlap with the oxygen atoms but only those forming a linear $\hbox {Co}^{+2}$–O–$\hbox {Cd}^{+2}$ bond. Small differences in the $B_{hf}$ values can be due to defects near Cd atoms or small distortions in the linear bond resulting in angles slightly different from 180$^{\circ }$ at low temperature.

**Figure 3:**
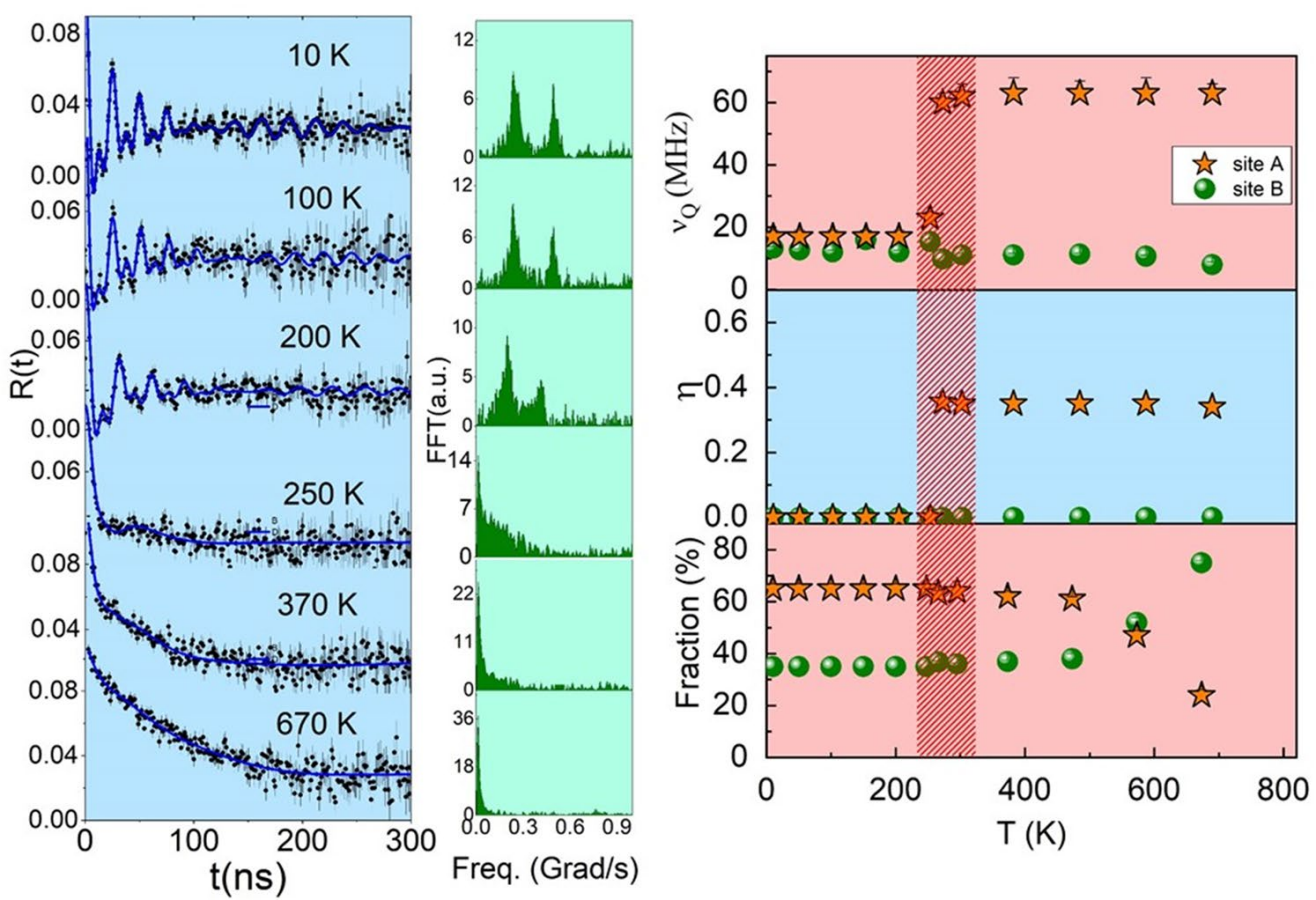
(**a**) PAC spectra and corresponding fast Fourier transform (FFT) measured with $^{111}$Cd in sample S2 at different temperatures. Continuous lines represent the fit of theoretical functions to experimental data. Note that above 250 K no modulation of PAC spectra was observed. (**b**) Temperature dependence of the quadrupole coupling frequency ($\nu _Q$), asymmetry parameter ($\eta$) and site fractions occupied by $^{111}$Cd probes. Arrow lines are guide to the eye indicating the sequence of temperature. PAC spectra and hyperfine parameters between the 10–295 K range are representative of sample S1

Figure 3 displays the spin-rotation spectra and the corresponding fast Fourier Transform (FFT) within a wide range of temperatures. It is important to stress that PAC measurements on sample S2 were taken with increasing temperatures from 10 to 670 K. All spectra taken in the 10–250 K range were fitted with the same two-site-fraction model described above: the major fraction corresponding to site A (external layer of NPs) and the minor fraction to site B (internal region of NPs). Results of the fit show that spectra from 250 K to 670 K present the site B with low-frequency $\nu _{QB}$, which corresponds to probe nuclei in a regular cubic environment (no modulation of the spectrum was observed). The temperature dependence of $\nu _{QB}$ shows a step around 250 K with a reduction in its value and a subsequent minor decrease with temperature, due to the thermal expansion of the lattice, whereas the asymmetry parameter vanishes for over the whole temperature range. The abrupt change in $\nu _{QB}$ is related to the known tetragonal distortion in CoO when the magnetic ordering is established, and it is associated with the contraction along the *c* axis with a possible deviation of the 3*d* radial function from a spherical distribution^38^. Therefore, the contraction along the c axis is expected to induce an increase in $V_{zz}$ (and $\nu _{Q}$), as it was experimentally observed and it is displayed in Fig. 3. It is noteworthy that previous PAC measurements of the electric field gradient (EFG) in CoO using $^{111}$Cd as probe nuclei could not detect the change at the ordering temperature^37,39^.

The evolution of the hyperfine parameters at the site A with temperature does not allow an unambiguous interpretation. At low temperatures, $\nu _Q$ and $\eta$ are similar to those of site B, though with a broader frequency distribution. At the ordering temperature and above, $\nu _Q$ and $\eta$ increase intensively, indicating that the presence of oxygen vacancies near the surface has a strong influence on the local environment of cation sites. The population of probe nuclei at site A decreases significantly for temperatures above 500 K due to the temperature-induced structure ordering near the surface of particles.

In particular, the spectrum at 250 K shows a sharp decrease in the initial amplitude within a few nanoseconds (ns) indicating an interaction with relatively high frequency. This effect can be ascribed to either a broad quadrupole frequency distribution or to the onset of a magnetic interaction due to a strong disorder. Nevertheless, below 250 K a clear magnetic dipole contribution appears but with a damped amplitude for all spectra.

**Figure 4:**
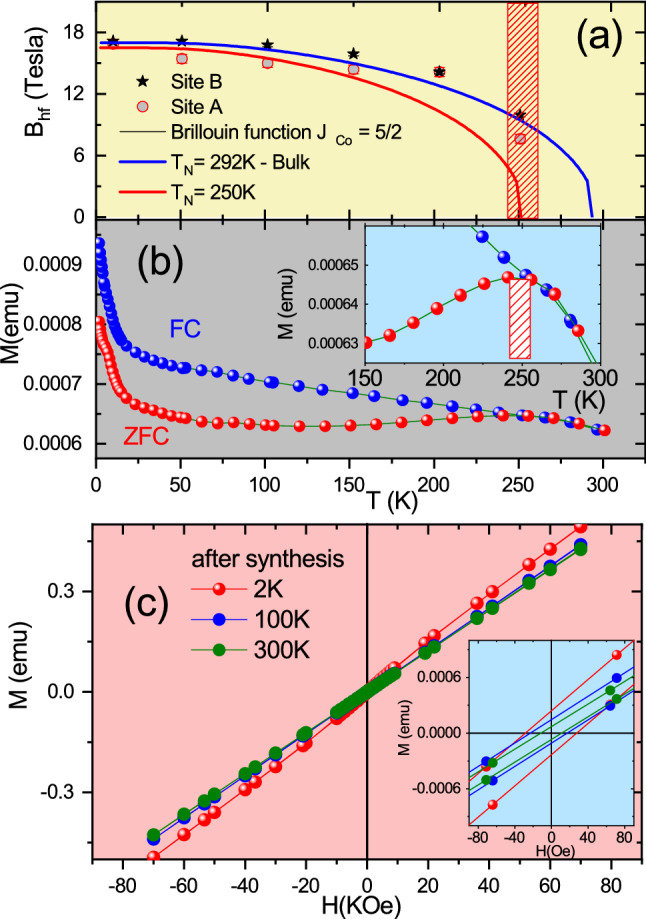
(**a**) Temperature dependence of $B_{hf}$ at sites A and B measured with $^{111}$Cd for sample S2 up to room temperature. The solid curves represent the Brillouin function for spin 5/2 ($B_{hf}$ data in this temperature range are representative of sample S1). The inset shows the maximum of the ZFC curve. (**b**) $M\times T$ curves and (**c**) *M*–*H* curves for sample S1. The inset in (**c**) shows the coercive field

Figure 4a displays the temperature dependence of $B_{hf}$ for both A and B sites determined from the fit to PAC spectra measured at temperatures below 250 K in S2. Results show that $B_{hf}$ of site A (corresponding to the major fraction) has a clear tendency to show a magnetic behavior with ordering temperature below 290 K as indicated by the solid curve that represents the Brillouin function for *S* = 5/2 fitted to the experimental values. On the other hand, the values of $B_{hf}$ for site B show a tendency for higher ordering temperature, which is estimated to be above 300 K. The antiferromagnetic ordering of small nanosystems of CoO with the Wurtzite-hexagonal structure (hcp-CoO phase) is $T_N$ $\sim$ 245 K^27,29^. Since the value of $T_N$ observed for sample S2 is higher, it was not possible to discard the occurrence of this phase.

In turn, results from the temperature dependence of ZFC–FC magnetization with an applied field of 100 Oe (see Fig. 4b) for sample S1 show a divergence between ZFC and FC curves below 250 K, and around this temperature it has a broad peak in ZFC curve, which could represent both Néel and blocking temperature. As it is known, blocking temperature is a characteristic of superparamagnetic materials. Our results from S1 sample do not present a magnetization process with no irreversibility, zero coercivity and a Langevin-like magnetic response in the M vs H curve above the ZFC–FC divergence at 250 K (see Fig. 4c), characteristics required for a superparamagnetic material^40,41^. Furthermore, comparing these results with PAC results, one can conclude that the divergence between ZFC and FC is more likely to represent Néel temperature. The non-typical behavior of an antiferromagnetic material and the broad peak is likely due to the wide size distribution of the nanoparticle diameter as revealed by TEM image. Moreover, the blocking temperature depends on external field applied^41^. Other interesting characteristic on ZFC–FC curves is the behavior at low temperature; our results show a pronounced increase of magnetization, which can be called paramagnetic tails. As discussed above, and shown by PAC results, small nanoparticles can present several types of defects on their surface, such as atomic vacancies, changes in the atomic coordination and amorphous phases, that may produce a magnetic disorder or uncompensated spins and contribute to the paramagnetic tails^28,34,42,43^.

Figure 4c displays the *M*–*H* hysteresis curves for S1 measured at low temperatures. One can clearly observe a linear dependence of magnetization for all measured temperatures, characteristics of antiferromagnetic or paramagnetic behavior contribution. The inset of Fig. 4c also shows a small coercive field of about 27 Oe at 2 K. This coercive field is likely due to uncompensated spins on the particle’s surface similar to those previously observed for CoO and NiO^30,44^. This magnetic behavior is in agreement with the PAC spectra which show a significant frequency distribution for all *R*(*t*) spectra suggesting strong surface-disorder effects. Therefore, the results allow us to conclude that the NPs present a high degree of spins and crystal surface disorder and PAC measurements are able to detect and quantify it through the site fraction determination. Moreover, the evolution of hyperfine parameters with temperature presents a strong evidence for the possible coupling between the magnetic and electrical properties.

After annealing, the S2 sample was placed in a closed-cycle refrigerator to carry out new PAC measurements at low temperatures. The spin rotation spectra were recorded starting from 10 K up to room temperature. All *R*(*t*) spectra were fitted using a model considering that probe nuclei occupy two site fractions. For example, the spin rotation spectra at 10 K (see results in Fig. S2a in the ESI) was fitted using the following two fractions: the site B (major fraction), corresponding to probe nuclei located at the core of nanoparticle at substitutional cation sites, and site A (minor fraction) was attributed to the probe nuclei at a region near the nanoparticle surface (see Fig. S2b in ESI).

**Figure 5:**
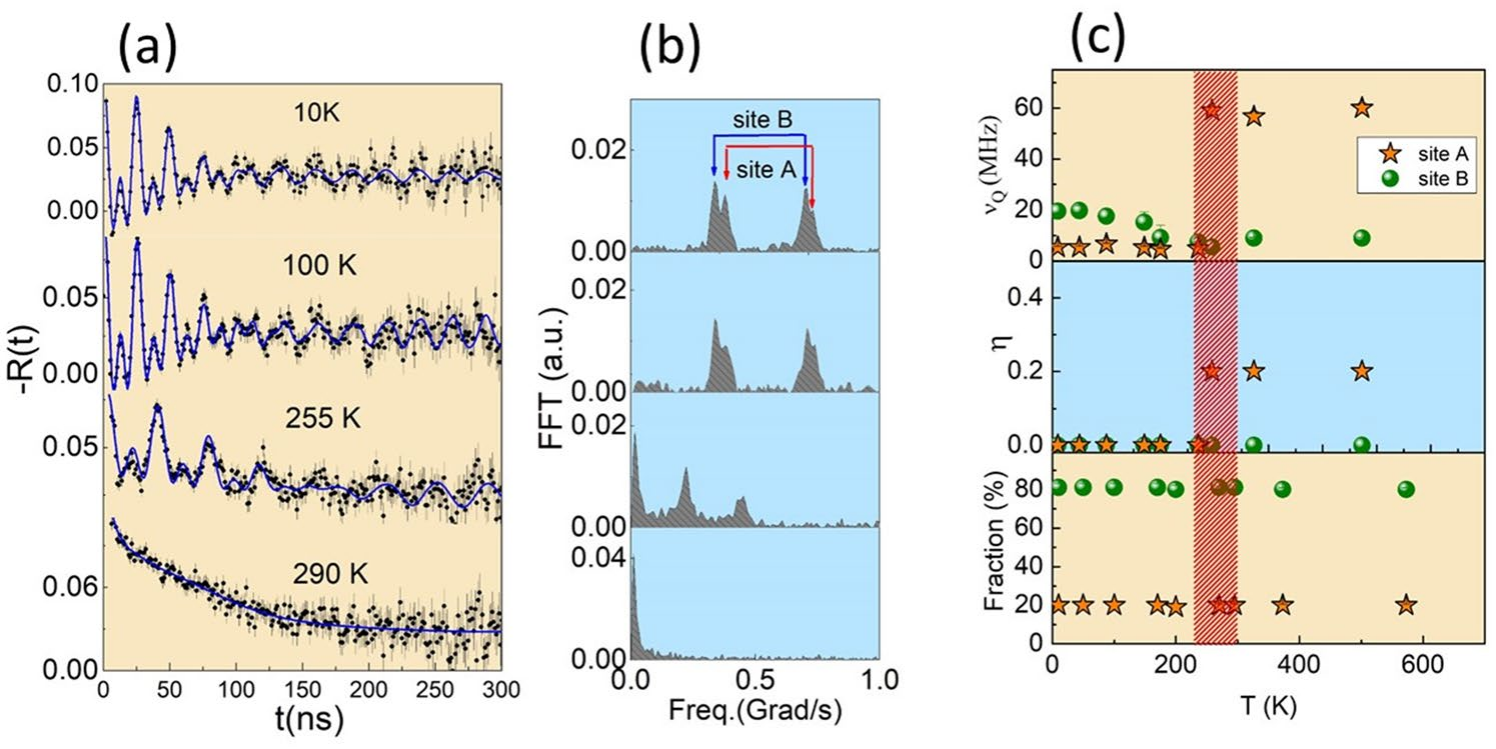
(**a**) Spin Rotation spectra measured with $^{111}$Cd in S2 sample of CoO after annealing (left) and respective Fast Fourier Transforms (right). The continuous lines represent the fit of theoretical functions to experimental data. (**b**) Temperature dependence of the quadrupole coupling frequency ($\nu _Q$), asymmetry parameter ($\eta$), and site fractions occupied by $^{111}$Cd probes

PAC spectra and their corresponding FFT for some temperatures within a wide range are shown in Fig. 5. In contrast to the results obtained for S2 after synthesis, PAC spectra show well-defined magnetic frequencies with good resolution FFT peaks characterizing the two magnetic interactions ascribed to site A and site B.

The well-defined frequencies in spin rotation spectra *R*(*t*) is a signature of probe nuclei in a regular crystalline environment which can be attributed to substitutional cation sites in the crystalline lattice of NPs due to the temperature-induced increase in their size from 6.5 nm to 22 nm during annealing procedure. As shown in Figs. 1d and S1b of ESI, a large orientated nanocrystal was observed in the TEM images after the annealing of S2. This result indicates a kinetic growth mechanism enhanced by the heating rate as reported in previous works^27,30,45,46^. Specifically, the growth of CoO nanoparticles is dependent on two mechanisms: a) Co diffusion into the remaining organic coating, into the lattice and intra lattice, and, principally, b) by coalescence and orientated attachment of CoO nanoparticles. It is noteworthy that, in the case of coalescence, there is no particular preference for the attachment whereas for the orientated attachment, one observes a common crystallographic alignment^47^. In addition, the heating to higher temperatures is accompanied by the relaxation of the lattice and a consequent kinetic diffusion of $^{111}$In($^{111}$Cd) probe nuclei into nanoparticles.

Differently for small nanoparticles (sample S1 and S2 after synthesis), the *R*(*t*) spectra above 250 K clearly show magnetic dipole interaction up to the transition temperature $T_N$ = 290 K. This temperature behavior is characteristic of fcc-CoO antiferromagnetic ordering. Therefore, we can conclude that the broad frequency distribution observed in PAC spectra that is attributed to surface effects in the small-size nanoparticles of S2 after synthesis was reduced after the annealing due to the increase in size and crystallinity of NPs. The contribution of surface effects to the modulation of spin rotation spectra in $\hbox {Fe}_3\hbox {O}_4$ NPs was studied with PAC spectroscopy by Effenberger et al. using $^{111}$In ($^{111}$Cd) probe nuclei^20^. Additionally, an anomalous local behavior of the $\nu _Q$ around the magnetic transition temperature similar to that observed before annealing is also present (see Fig. 5b). The difference is that after annealing the population of site fractions ($\sim$ 80% for site B and $\sim$ 20% for site A) remains the same of those observed for 670 K during the annealing (see Fig. 3b). It was suggested that the temperature dependence of EFG tensors could give information on the onset of charge or orbital ordering and support the idea of local tetragonal distortion of oxygen coordination emerging below the magnetic transition temperature, which can be correlated with short-range magnetic interactions^38^. Similarly to small particles (6.5 nm), the hyperfine data support the possible coupling between the magnetic and electrical properties below transition temperature (see Figs. 3b, 5b).

**Figure 6:**
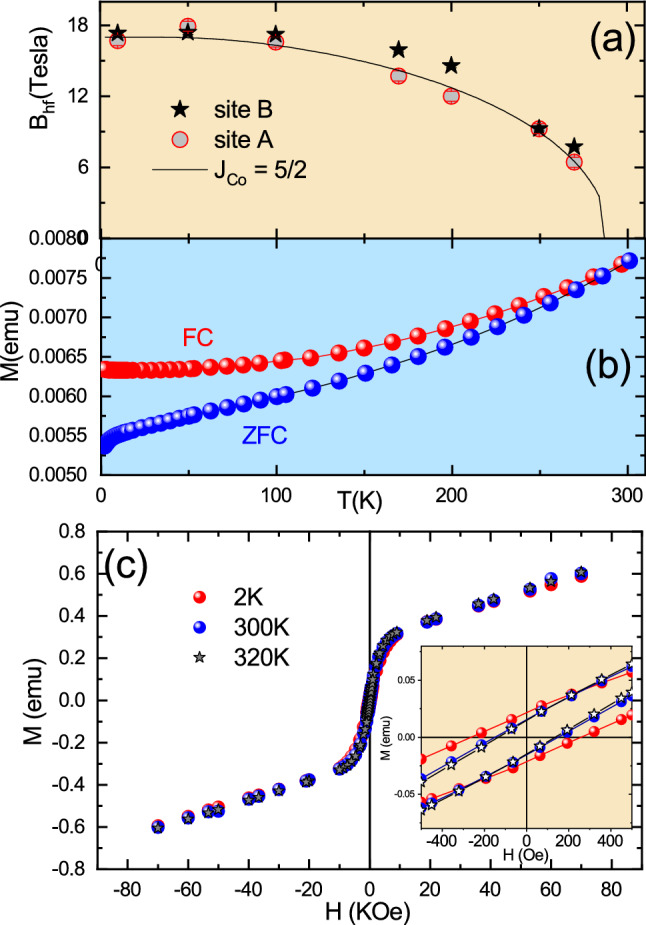
(**a**) Temperature dependence of $B_{hf}$ measured with $^{111}$Cd for sample up to room temperature. (**b**) $M\times T$ curves and (**c**) *M*–*H* curves for sample S2 after annealing. The inset in (**c**) shows the coercive field

Figure 6 displays $B_{hf}$ as a function of temperature together with the results of magnetization measurements for sample S2 after annealing. The behavior of $B_{hf}$ as a function of temperature for both sites A and B is consistent with the CoO antiferromagnetic interactions with Néel temperature around $T_N$ $\sim$ 290 K, which characterizes the rock-salt antiferromagnetic structure. The magnetic dipole interaction at the lowest temperature for site A with site fraction population $f_A$ = 20% is characterized by $B_{hf} = 17.1 (2)$ T plus an electric quadrupole interaction with quadrupole frequency $\nu _Q = 5(1)$ MHz with vanishing asymmetry parameter ($\eta _B = 0$). Above 250 K, $\nu _Q$ $\sim$ 60 MHz and $\eta _B = 0.2$, suggesting local distortion at the surface region. On the other hand, at site B with population $f_B = 80\%$ a similar behavior for the magnetic dipole interaction was observed with $B_{hf} = 17.3(2)$ T, quadrupole frequency $\nu _Q = 19.5(3)$ MHz, $\eta = 0$ and an angle $\beta = 30^{\circ }$ between $B_{hf}$ and the $V_{zz}$. This quadrupole interaction may be associated with local distortions and the origin of the tetragonal deformation of CoO has been debated and attributed to the magnetostrictive effect of the spin-orbit coupling below the magnetic ordering temperature^48,49^, whereas another work suggests that the origin is due to the Jahn-Teller distortion^50^.

Our results show that the structural tetragonal distortion of the $\hbox {CoO}_6$ octahedra in CoO with a contraction along the c axis is noticeable by PAC measurements with $^{111}$Cd probe nuclei with an expected increase in $V_{zz}$ at temperatures below $T_N$. It is important to mention that the PAC technique is sensitive to local changes and has been applied to several studies such as perovskite-type oxides and multiferroic materials in which electronic distortions are predominant^51–54^.

The occurrence of angle $\beta = 30^{\circ }$ between $B_{hf}$ (along the magnetic moment direction) and $V_{zz}$ was observed using other techniques such as neutron diffraction measurements in CoO single crystal where an angle of 27$^{\circ }$ between the *c*-axis of the crystal and the magnetic moment at Co atoms has been reported^55,56^. Below transition temperature, the angles were between 59$^{\circ }$ and 44$^{\circ }$ for powder samples^57^. A good example of PAC spectroscopy technique sensitivity to the direction of $B_{hf}$ with respect to $V_{zz}$ has been noted in the studies of thin film surfaces: terrace, interfaces, incorporated-step and ultrafine films where the EFG values may change in near-interfacial regions^58^.

The ZFC–FC curves in Fig. 6b do not show any indication of temperature transition ($T_N$) and blocking temperature ($T_B$) up to 300 K. In contrast with the sharp increase in the ZFC–FC curves at low temperature observed for small nanoparticles (displayed in Fig. 4), the ZFC curve shows a decrease in M values at very low temperatures, whereas the FC curve remains practically unchanged below 50 K. This difference is probably a consequence of the thermal treatment that resulted in the reduction of the amount of capping material, the presence of defects, internal voids and strains. On the other hand, it was observed that the *M*–*H* curve for sample S2 presents an Langevin-like shape, a fingerprint of the ferromagnetic coupling, in the hysteresis cycle (see Fig. 6c). In addition, the behavior of the curve even for high fields does not show any tendency to saturate. This is an indication of the presence of a strong competition between ferromagnetic interactions and frustrated magnetic state interactions.

Since X-ray diffraction analysis also supported by hyperfine parameters has shown that the CoO NPs have the fcc crystal structure and, therefore, would order antiferromagnetically, what is the origin of the ferromagnetic behavior? In fact, the ferromagnetism at room temperature is a topic of intense discussion by the academic community and two interpretations are the most probable in the case of CoO ferromagnetism. In the first, the ferromagnetic behavior at room temperature has been attributed to “uncompensated” and “frustrated” surface moments on the nanoparticle, this fact is a consequence of reduced surface coordination, called by the Néel theory^30–33,59^. Also, this uncompensated spin moment may be formed due to the presence of a thin $\hbox {Co}_2\hbox {O}_3$ layer on the surface of CoO, and the Coulomb repulsion, due to the charge transfer process of $\hbox {Co}^{3+}$ to $\hbox {Co}^{2+}$ at CoO/$\hbox {Co}_3\hbox {O}_4$ interface, has been suggested by Li et al. as the origin of the ferromagnetic behavior^60^. However, in our work we discard the presence of the thin layer of $\hbox {Co}_2\hbox {O}_3$ because its phase has shown temperature transition around 40 K and this fact was not observed by our magnetic measurements^28,61^.

In the second interpretation, the occurrence of ferromagnetism is attributed to the presence of metallic cobalt contributions formed during the thermal treatment, as in the case studied in this paper due to the annealing in a helium atmosphere until 670 K. During the annealing, a diffusion mechanism of Co atoms may be involved allowing the occurrence of probable dilute Co clusters. In fact, the presence of low intensity peaks in the X-ray patterns as shown in Fig. 2c within the range of 2$\theta$ $\sim 45^{\circ }$–$48^{\circ }$ corresponding to metallic hcp cobalt structure indicates to the presence of the metallic cobalt. Complementary, as previously reported, CoO phase may be reduced to metallic Co at high temperature in vacuum yielding Co/CoO interface in the nanoparticles^36,45^. Therefore, the presence of metallic Co in sample S2 after annealing is likely to be associated with site A.

Another point is that even above room temperature (300 K), the S-like magnetic behavior is present with coercive field ($H_c = 149$ Oe) whereas at 10 K, $H_c = 260$ Oe, which is characteristic of the ferromagnetic contribution probably due to the presence of metallic cobalt. It is worth noting that the transition temperatures for both hcp and fcc metallic phases are approximately 680 K and 1030 K, respectively. For the samples before annealing, taking PAC fractions as absolute one would see the 2-cell-thick “shell”. It is unlikely to be “visible” on TEM. Nevertheless, we believe that during annealing procedure, dilute small clusters of magnetic metallic Co were formed on the surface or into CoO nanocrystals. The PAC spectroscopy is a highly-sensitive local technique that requires the incorporation of a probe nuclei into the crystalline lattice of the studied sample. PAC results for the sample S2 only show hyperfine fields compatible with CoO, as described above, which strongly indicates an affinity of $^{111}$Cd with fcc-CoO phase. The magnetic hyperfine fields for metallic cobalt were reported to be $- 16.4$ T, $- 28.6$ T, and $- 23.7$ T, respectively for bcc, hcp, and fcc structures when using the $^{111}$Cd nuclei probe^62^, but these fields were not observed in this experiment. Reinforcing this conclusion, measurements in the metallic phases using PAC spectroscopy were only possible by introducing the parent nucleus by ion implantation methods^62^; because these metallic phases have a high packing density which makes it difficult to introduce the probes to the metal particles.

## Discussion

As previously mentioned the magnetic transition temperature is a very important parameter for nanoparticles and often it is not clearly determined from magnetic techniques due to superposition of blocking temperature. PAC measurements with $^{111}$Cd at Co sites is a valuable technique in order to identify the magnetic transition temperature for CoO NPs just after synthesis and after annealing at two site fractions ascribed to the core and surface of particles. In these NPs with different sizes it was possible to observe contributions from two magnetic interactions that are predominant with temperature transition around 250 K and 290 K at 6.5 nm and 22.1 nm, respectively. It is noteworthy that this low transition temperature around 250 K for the 6.5 nm particles is due to the competition of interactions between the core and the surface of the CoO nanoparticles which would present a lack of compensation due to structural and magnetic disorder in the region near the surface which is evidenced below 250 K.

Moreover, a predominant fcc CoO structure with additional low intensity ($\sim$ 0.8%) peaks, attributed to the presence of dilute metallic hcp Co clusters, was observed in the XRD pattern for the 20 nm NPs formed after annealing. PAC results show no magnetic dipole interactions above room temperature for this sample in agreement with paramagnetic interactions at fcc CoO structure. These facts allow us to conclude that the $\hbox {Cd}^{+2}$ probe ion has a strong affinity with CoO rock-salt (fcc). Reinforcing this conclusion, measurements in the metallic phases using PAC spectroscopy were only possible by introducing the parent nucleus by ion implantation methods; because these metallic phases have a high degree of packing which makes it difficult to locate the probe nuclei inside the metal particles.

Finally, an anomalous temperature dependence behavior of electric hyperfine parameters were observed at temperatures around the magnetic ordering transition. The temperature dependence of the quadrupole frequency clearly indicates a crystalline distortion. Furthermore, it is suggested that the temperature dependence of EFG tensors can give information on the onset of charge or orbital ordering and support the idea of local distortion of oxygen coordination with short-range magnetic interactions in agreement with other reports. In conclusion, hyperfine interactions measured by PAC with $^{111}$Cd as probe nuclei is a sensitive tool to locally characterize and investigate magnetic properties, formation of dilute impurity clusters and slight crystalline structural distortions.

## Methods

$^{111}$Cd is one of the most used probe nuclei for PAC measurements due to optimal nuclear properties as well as due to the atomic properties of the radioactive $^{111}$In parent nucleus. There are different routes to introduce radioactive nuclei as dilute impurities into matrices like nanoparticles, thin films or bulks. In the present work, radioactive $^{111}$In nuclei were then introduced into samples during the CoO-NPs synthesis.

### Synthesis of nanoparticles

Samples of CoO nanoparticles were synthesized by the thermal decomposition method described by Effenberger et al.^20^. In this synthesis, 2 mmol of Cobalt (II) acetylacetonate were dissolved in 20 ml of diphenyl ether, 4 mmol of oleylamine, 6 mmol of oleic acid and 10 mmol of 1,2-octanediol. A three-neck flask was used to mix and homogenize the reagents using an ultrasonic cleaner equipment. The flask was connected to a condenser and the mixture was heated at 258 $^{\circ }$C for 2 h under nitrogen atmosphere. After cooling to room temperature, ethanol (proportion of 1:3) was added to the solution, and then the nanoparticles were precipitated and separated through centrifugation at 5000 RPM per 30 minutes. This last process was repeated three times until the liquid solution became clear, in order to remove the excess of organic material. The precipitate was dried under low pressure (about 1 kPa) per 24 h and a resulting powder was obtained. To perform PAC measurements a small volume of radioactive $^{111}\hbox {InCl}_3$ was added to the initial solution during the preparation of nanoparticle samples.

### Characterization of nanoparticles

**Figure 7:**
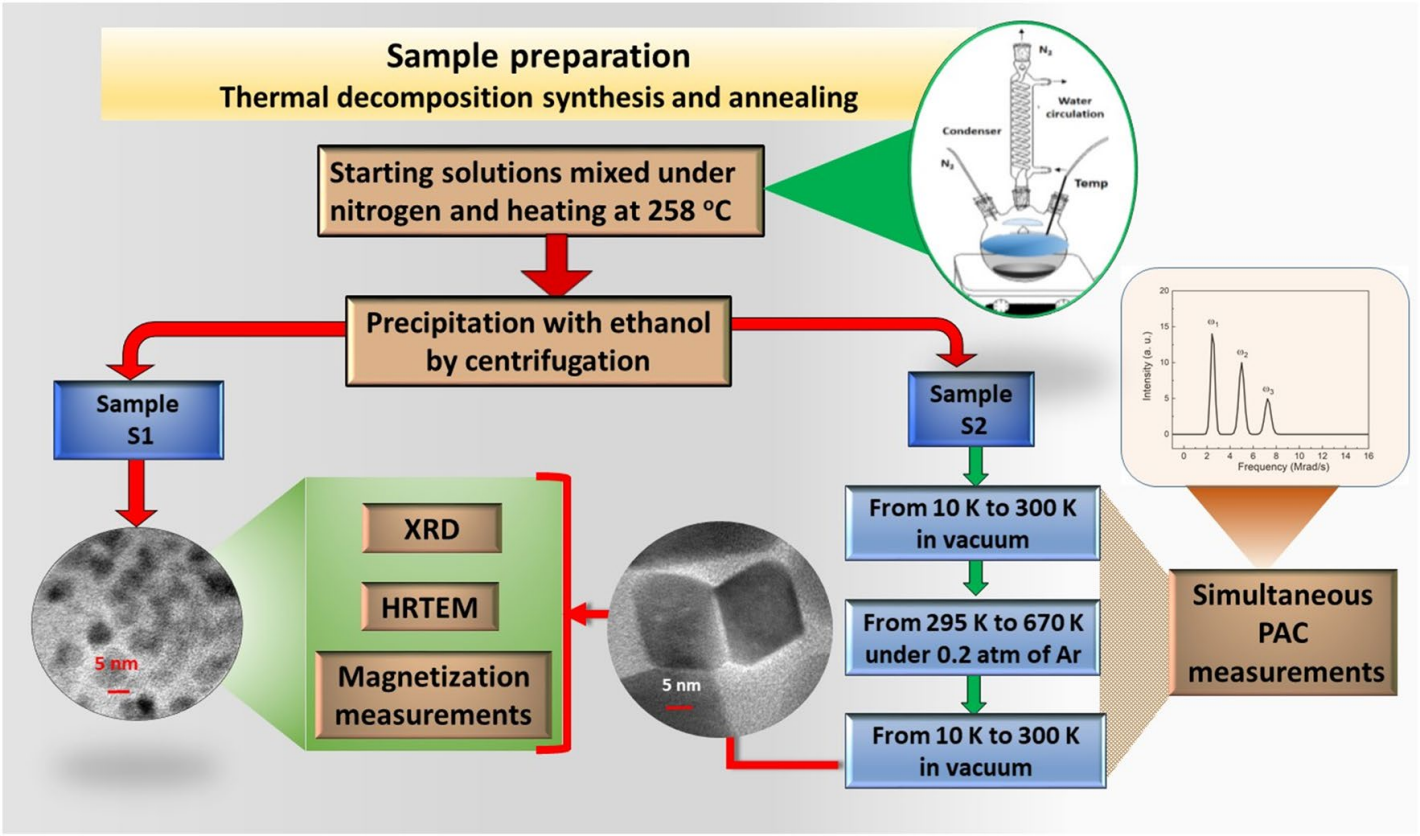
Scheme displaying the steps of the samples preparation and characterization by XRD, TEM and magnetization measurements of S1 and S2 as well as the measurements by PAC before (at temperatures below RT) and during the steps of the annealing of S2. PAC measurements in the 10–300 K range after synthesis are representative of sample S1 (see text)

After the synthesis, the resulting powder sample was separated in two parts. One part, hereafter called sample S1, was used to check the structural and magnetic properties of the particles after the synthesis procedure. The second part, hereafter called sample S2, was placed in the sample holder of the He closed-cycle refrigerator device to be measured at different temperatures in a range from 10 K to room temperature (RT) using the PAC spectrometer with four-$\hbox {BaF}_2$ detectors of the Hyperfine Interactions Laboratory in the ‘Instituto de Pesquisas Energéticas e Nucleares’ (IPEN) in São Paulo, Brazil. These measurements were carried out to investigate the behavior of as-synthesized CoO nanoparticles at low temperatures, therefore, all PAC spectra and hyperfine parameters obtained in the 10–295 K range are representative of sample S1. After measurements below RT, the sample S2 was sealed in a quartz tube under low pressure of argon (around 0.2 atm) and placed inside a small furnace to be measured in the PAC spectrometer while being annealed. This annealing followed by PAC measurements started at room temperature with subsequent steps with temperature being increased of 100 K after each one until reaching 670 K. Each step had a dwell time of 12 h during which the spin rotation spectra were acquired. After this annealing, the sample S2 were cooled down to RT (during which, PAC measurements were carried out at 573 K and 373 K) and had PAC spectra taken at different temperatures in the range from 10 to 300 K in the He closed-cycle refrigerator. These measurements were carried out to investigate the behavior of CoO nanoparticles at low temperatures after the annealing. The sample S2 was then stored until the activity was low enough to be considered as background radiation. The size and morphology of samples S1 and S2 were then checked by transmission electron microscopy (TEM) while the crystalline structure was analyzed by X-ray diffraction (XRD). The magnetic characterization of both samples S1 and S2 was performed by magnetization measurements with field cooling (FC) and zero field cooling (ZFC) in the temperature range from 2 to 300 K as well as magnetization as a function of the applied field. Figure 7 presents a schematic sequence of measurements and annealing of both samples after synthesis. A brief description of the PAC technique as well as the main equations involving the hyperfine parameters are described below, further details of the technique can be found elsewhere^63^.

### Perturbed angular correlation technique: a brief overview

The method is based on the conservation of angular momentum between the spin direction of the intermediate level of a $\gamma$-emitter probe nucleus which emits two gamma radiations, $\gamma _1$ and $\gamma _2$ in cascade, and the direction of the $\gamma _2$ emission pattern. The hyperfine interaction consists of the coupling of the total angular momentum (*J*) of the electrons with the spin (*I*) of the nuclear intermediate level. When the probe nucleus is inserted into a material, the hyperfine interaction induces a time variation of this emission pattern and its measurement in a plane at different angles permits obtaining the components of the electric field gradient (EFG) tensor and/or the intensity of the magnetic hyperfine field ($B_{hf}$). Combined electric plus magnetic hyperfine interactions can also be measured. The number of radioactive nuclei necessary to perform experiments is generally less than 10$^{12}$, which in most cases represents a highly diluted concentration in the range between 0.01 and 1 ppm. This number of atoms is still enough to produce an observable modulation of the decay curve of the coincidence function obtained from the $\gamma _1$ and $\gamma _2$ simultaneous detection. The ratio of these functions measured with detectors at 90$^{\circ }$ and 180$^{\circ }$ is described by the so-called anisotropy ratio (*R*(*t*)) given by $R(t) = \sum _i A_{22} f_i G_{22}^i$. A fit to the *R*(*t*) function, also usually referred as spin-rotation function, allows identifying multiple fractions ($f_i$) of probe atoms surrounding by different local environments in which these probes interact with the charge and spin density through hyperfine interactions given by respective perturbation functions ($G_{22}^i (t)$). $A_{22}$ is the angular correlation coefficient, which for $^{111}$In($^{111}$Cd) is $A_{22} = -0.18$^64^. Because $A_{22}$ is negative, the spin-rotation spectra for $^{111}$Cd is displayed as $-R(t)$ with the y axis inverted.

For electric quadrupole interactions, the perturbation function is given by $G_{22} = S_{20} + \sum _{n=1}^3S_{2n}(\eta )cos(\omega _n t)exp(-\delta _n \omega _n t)$, with the transition frequencies $\omega _n$ being related to the quadrupole frequency $\nu _Q = (eQV_{zz})/h$ by $\omega _n = g_n (\eta )\nu _Q$, where *Q* is the nuclear electric quadrupole moment of the intermediate level of nuclear probe, and the exponential factor accounts for a Lorentzian distribution of relative width $\delta _n$ on the transition frequencies $\omega _n$. The coefficients $g_n(\eta )$ and $S_{2n}(\eta )$ are functions of the asymmetry parameter $\eta = (V_{xx} - V_{yy})/V_{zz}$ , where $V_{kk}$, with k = x,y,z, denotes the principal component of the electric field gradient (EFG) tensor. Since *Q* of the probe nucleus is well known, the experimental determination of $\nu _Q = eQV_{zz}/h$ allows the calculation of the major component ($V_{zz}$) of EFG as well as the measure of $\eta$ provides information about the configuration of the electric field gradient in the crystallographic site where the probe nuclei are localized. The parameter $\eta$, with values limited to $0< \eta < 1$, thus measures the deviation of the local charge distribution from axial symmetry due to local distortion of the crystallographic site. Hence, it is important to stress that $\eta$ gives information on the local symmetry around the probe nucleus.

The perturbation function for a pure magnetic interaction is given by $G_{22} (t) = 0.2 + 0.4\sum _{(n=1)}^2cos(n\omega _L t)exp(-\delta \omega _L t)$, where $\omega _L$ is the Larmor frequency and its experimental determination allows the calculation of the magnetic hyperfine field $B_{hf}$ since $\omega _L = g\mu _N B_hf/\hbar$, where $\mu _N$ is the nuclear magneton and *g* is the nuclear g-factor. In combined electric quadrupole plus magnetic dipole interactions, the angle ($\beta$ between directions of $V_{zz}$ and $B_{hf}$ can also be determined. Details regarding the experimental methodology, particularly PAC measurements on metal and oxides, can be found elsewhere^51,65–68^. Radioactive nuclei are added to samples to be measured by PAC through several methods. Ionic implantation and thermal diffusion are two usual methods^69,70^. Thermal diffusion, by its nature, requires samples to be heated at high temperature for long period. Ionic implantation usually needs a rapid thermal annealing at very high temperature for a short time to minimize the irradiation damage in the crystalline structure due to collisions with ions. Both methods are, therefore, not suitable for introduction of radioactive nuclei in nanoparticle samples, since high temperatures may modify the shape and size of particles. Additionally, ion implantation requires nanostructured systems with minimum sizes greater than one hundred nm, depending on the energy of the implanting ions otherwise the radioactive ions crosses the system without stopping inside it. $^{111}$Cd yielded by the electron-capture decay of $^{111}$In, with the subsequent emission of successive gamma-rays (171 KeV and 245 keV) is one of the most used probe in PAC technique due to the optimal nuclear properties of both the parent ($^{111}$In) and the daughter ($^{111}$Cd) as well as the electronic configuration of the Cd ion.

## Supplementary information

Supplementary Information.
