# Supplementary material for: Crystalline and magnetic properties of CoO nanoparticles locally investigated by using radioactive indium tracer
Source: Sci Rep. 2021 Oct 25;11:21028. doi: 10.1038/s41598-021-99810-y (PMC8546082; doi:10.1038/s41598-021-99810-y)
Supplement: Supplementary file 1 — Supplementary Information. [file 41598_2021_99810_MOESM1_ESM.pdf]

Supplementary Information

Crystalline and magnetic properties of CoO nanoparticles locally investigated by using radioactive indium tracer

Renata V. Santos[1], Gabriel A. Cabrera-Pasca[1,2], Cleidilane S. Costa[1,2], Brianna Bosch-Santos[3], Larissa Otubo[3], Luciano F. D. Pereira[3], Bruno S. Correa[3], Fernando B. Effenberger[3], Anastasia Burimova[3], Rafael S. Freitas[4], and Artur W. Carbonari[3,*]

[1]Programa de Pós-Graduação em Ciência e Engenharia de Materiais – PPGCEM- Universidade Federal do Pará, 67130-660 Ananindeua, PA, Brazil
[2]Faculdade de Ciências Exatas e Tecnologia, Universidade Federal do Pará, 68440-000 Abaetetuba, PA, Brazil
[3]Instituto de Pesquisas Energéticas e Nucleares, IPEN-CNEN/SP, 05508-000 São Paulo, SP, Brazil
[4]Instituto de Física, Universidade de São Paulo, 05508-090 São Paulo, SP, Brazil
[*]carbonar@ipen.br

1 X-RAY DIFFRACTION MEASUREMENTS.

Samples S1 and S2 had their crystalline structure checked by X-ray diffraction measurements which were carried out in a Philips X´Pert PRO PW 3040/00 diffractometer in the Engineering College of São Paulo University. The 2θ angle ranges from 10° to 100° with steps of 0.05^o taken during 400 s. Results are displayed in Fig. S1.

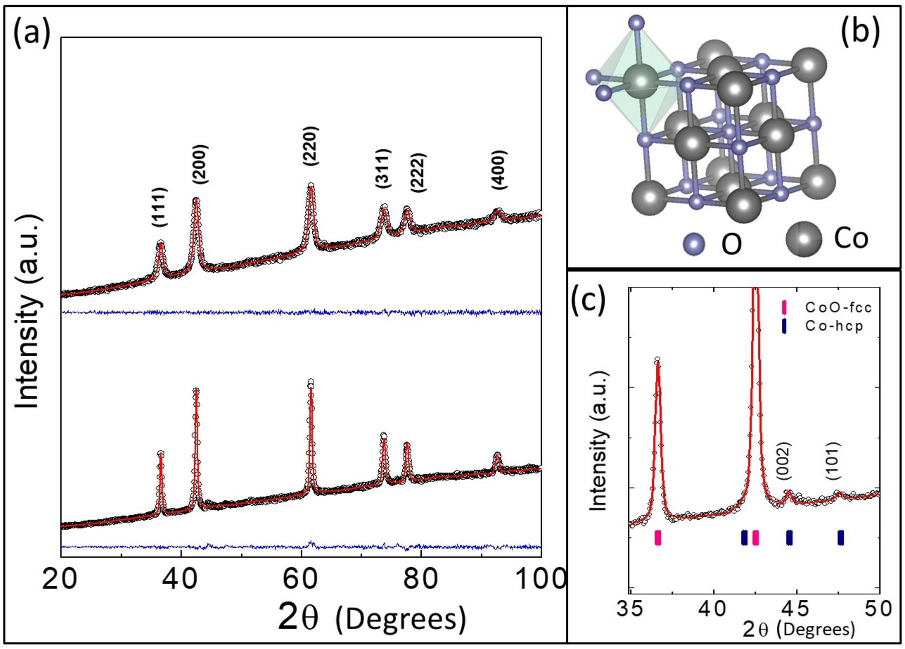

Figure S1: (a) XRD results for S1 sample (top) and S2 sample (bottom). (b) Crystalline structure of CoO where large spheres represent Co atoms and small spheres represent oxygen atoms. c) XRD pattern for S2 sample showing two extra peaks, (002) and (101), ascribed to hcp Co.

2 PERTURBED ANGULAR CORRELATION.

PAC results for sample S2 at 10 k as well as the TEM image of this sample is displayed in Fig. S2 below.

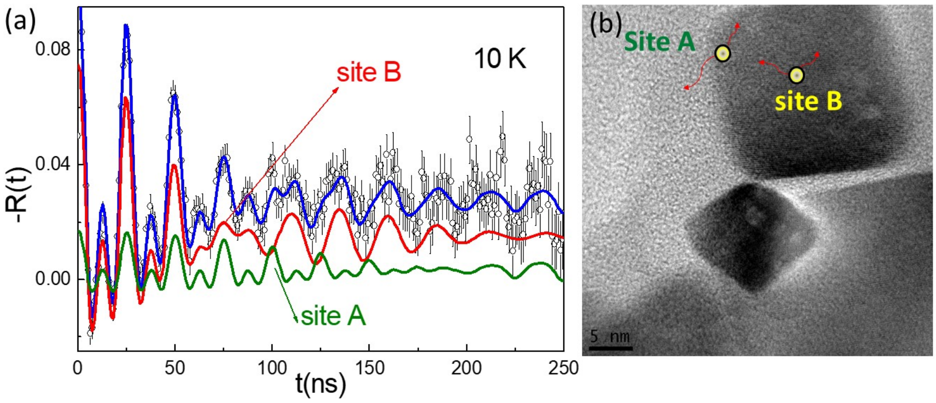

Figure S2: a) Spin rotation spectrum measured at 10 K for sample S2 after annealing. The continuous lines represent the fit of theoretical function to experimental data. b) TEM image of S2 sample with a representation of the localization of ^{111}Cd probe nuclei near surface called site A and into nanocrystal called site B.
